# Predictive Efficacy of Low Burden EGFR Mutation Detected by Next-Generation Sequencing on Response to EGFR Tyrosine Kinase Inhibitors in Non-Small-Cell Lung Carcinoma

**DOI:** 10.1371/journal.pone.0081975

**Published:** 2013-12-20

**Authors:** Hye Sook Kim, Jae Sook Sung, Song-Ju Yang, Nak-Jung Kwon, LiHua Jin, Seung Tae Kim, Kyong Hwa Park, Sang Won Shin, Han Kyeom Kim, Jin-Hyoung Kang, Jeong-Oh Kim, Jae Yong Park, Jin Eun Choi, HyoungKyu Yoon, Chan Kwon Park, Kap-Seok Yang, Jeong-Sun Seo, Yeul Hong Kim

**Affiliations:** 1 Division of Oncology/Hematology, Department of Internal Medicine, College of Medicine, Korea University, Seoul, Korea; 2 Cancer Research Institute, Korea University, Seoul, Korea; 3 Macroge Inc., Seoul, Korea; 4 Department of Pathology, College of Medicine, Korea University, Seoul, Korea; 5 Division of Medical Oncology, Seoul Saint Mary's Hospital, The Catholic University of Korea, Seoul, Korea; 6 Laboratory of Medical Oncology, Research Institutes of Medical Science, The Catholic University of Korea, Korea; 7 Lung Cancer Center, Kyungpook National University Medical Center, Daegu, Korea; 8 Department of Biochemistry, School of Medicine, Kyungpook National University, Daegu, Korea; 9 Division of Pulmonology, Department of Internal Medicine, Saint Mary's Hospital, College of Medicine, The Catholic University of Korea, Korea; 10 Genomic Medicine Institute (GMI), Medical Research Center, Seoul National University, Seoul, Korea; 11 Department of Biochemistry, Seoul National University College of Medicine, Seoul, Korea; 12 Department of Biomedical Sciences, Seoul National University Graduate School, Seoul, Korea; H. Lee Moffitt Cancer Center & Research Institute, United States of America

## Abstract

Direct sequencing remains the most widely used method for the detection of epidermal growth factor receptor (EGFR) mutations in lung cancer; however, its relatively low sensitivity limits its clinical use. The objective of this study was to investigate the sensitivity of detecting an epidermal growth factor receptor (EGFR) mutation from peptide nucleic acid-locked nucleic acid polymerase chain reaction (PNA-LNA PCR) clamp and Ion Torrent Personal Genome Machine (PGM) techniques compared to that by direct sequencing. Furthermore, the predictive efficacy of EGFR mutations detected by PNA-LNA PCR clamp was evaluated. EGFR mutational status was assessed by direct sequencing, PNA-LNA PCR clamp, and Ion Torrent PGM in 57 patients with non-small cell lung cancer (NSCLC). We evaluated the predictive efficacy of PNA-LNA PCR clamp on the EGFR-TKI treatment in 36 patients with advanced NSCLC retrospectively. Compared to direct sequencing (16/57, 28.1%), PNA-LNA PCR clamp (27/57, 47.4%) and Ion Torrent PGM (26/57, 45.6%) detected more EGFR mutations. EGFR mutant patients had significantly longer progressive free survival (14.31 vs. 21.61 months, P = 0.003) than that of EGFR wild patients when tested with PNA-LNA PCR clamp. However, no difference in response rate to EGFR TKIs (75.0% vs. 82.4%, P = 0.195) or overall survival (34.39 vs. 44.10 months, P = 0.422) was observed between the EGFR mutations by direct sequencing or PNA-LNA PCR clamp. Our results demonstrate firstly that patients with EGFR mutations were detected more frequently by PNA-LNA PCR clamp and Ion Torrent PGM than those by direct sequencing. EGFR mutations detected by PNA-LNA PCR clamp may be as a predicative factor for EGFR TKI response in patients with NSCLC.

## Introduction

Lung cancer is the leading cause of cancer-related deaths worldwide, and standard therapeutic strategies, including surgery, chemotherapy, and radiotherapy have reached a plateau [1]. Recently, pharmacological treatment of non-small cell lung cancer (NSCLC) has undergone a major change for patients with somatic mutations in the tyrosine kinase domain of the epidermal growth factor receptor (*EGFR*) gene. Patients with NSCLC harboring activating *EGFR* mutations benefit from treatment with tyrosine kinase inhibitors (TKIs) as compared to conventional cytotoxic chemotherapy [2]–[4]. Unfortunately, in spite of relatively consistent performance of *EGFR* TKIs in patients with *EGFR* mutations, patients with wild-type *EGFR* show various responses to *EGFR* TKIs [5]–[8].

Up until now, screening and identification of EGFR mutations have routinely carried out by direct sequencing. However, it is now a well-established fact that the sensitivity of direct sequencing is suboptimal for many clinical tumor samples, in that mutant DNA alleles must comprise over 25% of the total DNA signals to be readily detected [9]. Considering limited samples for mutational analysis in lung cancer such as small tissue biopsies or cytological specimens and high proportion of normal cells contained in these samples, low sensitivity of direct sequencing presents critical disadvantages. Therefore, the issue that substantial portion of patients who can benefit from EGFR TKIs might be lost due to limited sensitivity had been arisen. These considerations continue to drive the development and evaluation of new techniques for the detection of *EGFR* mutations [3], [9]–[13]. Among various sequencing techniques, the mutant-enriched polymerase chain reaction (PCR) is a rapid and sensitive assay which can detect on mutant gene among as many as 10^3^ to 10^4^ copies of the wild type gene. By amplification of a particular DNA sequence, mutant-enriched PCR can detect significant portion of mutations that could be missed by direct sequencing.

A number of next-generation sequencing (NGS) is being carried out, emphasizing short turnaround time and thorough outcome [14]. The emergence of comprehensive genomic profiling by NGS has led investigators to question whether more thorough gene-sequencing techniques could lead to the discovery of potential targets for relapsed or metastatic NSCLC. However, the efficacy of NGS for predicting clinical benefits from *EGFR* TKIs has yet to be fully established. The clinical significance of low-signal mutant in treatment with EGFR-TKIs and quantification of the mutation need further investigation to optimize treatment selection and strategy. Furthermore, the functional consequences of a small number of novel *EGFR* mutations need to be understood [15], [16].

In the present study, we evaluated the sensitivities of peptide nucleic acid-locked nucleic acid (PNA-LNA) PCR clamp and Ion Torrent Personal Genome Machine (PGM) compared to that of direct sequencing for detecting *EGFR* mutations. Additionally, we evaluated the clinical impact of considering an EGFR mutation positive as detected by more sensitive methods.

## Materials and Methods

### Tumor samples

Tumor samples were obtained from patients with NSCLC at five institutions in Korea. Study cohort A consisted of surgically resected or biopsy NSCLC samples enriched with adenocarcinoma histology. The purpose of analysis of cohort A was comparison of sensitivities of 3 different methods to detect *EGFR* mutations. PNA-LNA PCR clamp and Ion Torrent PGM were compared to direct sequencing. Study cohort B consisted of patients who received gefitinib or erlotinib during the treatment period. In study cohort B, clinical data for the NSCLC patients were searched retrospectically, including gender, age at diagnosis, tumor histology type, clinical staging, smoking status, and response to treatment. The purpose of analysis of cohort B was comparison of power to predict performance of EGFR-TKIs in terms of response rate, progression free survival (PFS) and overall survival (OS). The study was approved by the institutional review board of each hospital (Korea University Anam & Guro hospitals, Seoul & Yeouido St. Mary's Hospital, Kyungpook National University hospital). The medical records and radiographic images of the patients were then reviewed to evaluate their clinicopathologic characteristics, tumor responses, adverse effects, and survival outcomes using a predesigned data collection format. Written informed consent was obtained from all patients and their data were processed and stored according to the principles expressed in the Declaration of Helsinki.

### Genomic DNA extraction and direct sequencing

All clinical specimens consisted of DNA prepared from formalin-fixed paraffin-embedded (FFPE) tissue or frozen tissue from patients with lung cancer. Slides from FFPE were examined by two independent pathologists from different centers to validate existence of tumor cells. Specimens were sent to central lab (PNA-LNA PCR Clamp in Cancer Research Institute, Korea University, Seoul, Korea and direct sequencing and Ion torrent PGM in Macrogen Inc.) for preparation and extraction of DNA. Genomic DNA was prepared using the Gentra Puregene DNA Isolation Kit (Qiagen, Hilden, Germany). The purity of extracted DNA was examined with spectrophotometric measurement. *EGFR* mutations in exons 18, 19, 20, and 21 were detected by PCR-based direct sequencing. PCR amplification was performed with 50 ng of genomic DNA. The following primers (forward and reverse, respectively) were used: exon 19 (5′–TGTCATAGGGACTCTGGATCC–3′ and 5′–AGCAGAAACTCACATCGAG–3′), exon 20 (5′– ACCTTTGCGATCTGCACAC–3′ and 5′–CAGGAAGCCTACGTGATGG–3′), and exon 21 (5′–CTTGGAGGACCGTCGCTTG–3′ and 5′–CCACCTCCTTACTTTGCCTC–3′). DNA was amplified during 35 cycles of 94°C for 30 s, 61°C for 30 s, and 72°C for 30 s, followed by a 10-min extension at 72°C. Sequencing reactions were performed in an MJ Research PTC-225 Peltier Thermal Cycler using ABI PRISM BigDye Terminator Cycle Sequencing Kits with AmpliTaq DNA polymerase (FS enzyme) (Invitrogen, Carlsbad, CA), following the protocols supplied by the manufacturer. The samples were resuspended in distilled water and subjected to electrophoresis in an ABI 3730xl sequencer (Invitrogen, Carlsbad, CA).

### Peptide nucleic acid-locked nucleic acid PCR clamp (PNA-LNA PCR Clamp) method

The PNA-LNA PCR clamp method preferentially amplifies mutant sequences and thus detects mutations. The method utilizes PNA clamp primers and locked nucleic acid (LNA) probes. Five probes were used to detect mutations in exon 19 (E746-A750del [2235–2249del], E746-A750 [2236–2250], L747-E749 A750P [2239–2247 and 2248]), exon 20 (T790M), and exon 21 (L858R). The PNA-LNA PCR clamp method was performed by Koichi Hagiwara Laboratories Inc. (Saitama, Japan) [12]. Real-time amplification monitoring for the PNA-LNA PCR clamping was performed using Smart Cycler (Cepheid, Sunnyvale, CA). Control genomic DNA mixtures containing the mutant and the wild-type EGFR gene at ratios of 1∶1, 0.1∶1.0, 0.01∶1.00 and 0.0 were used to establish standards for the amplification curve. The technical threshold for a positive signal has been set at 1%, which means 1 mutant allele is detected in the presence of 100 wild-type alleles. Inversely, samples in which the cancer content is less than 1% result in negative outcome.

### Personal genome machine (PGM) sequencing using the Ion AmpliSeq™ Cancer Panel on the Ion Torrent

Ion Torrent sequencing was performed following the Ion Torrent protocol (Life Technologies). Whole genomic DNA was isolated from the frozen tissue of lung cancer patients using a Qiagen genomic DNA isolation kit (Qiagen, Hilden, Germany). Twenty micrograms of genomic DNA was amplified by the Ion AmpliSeq™ Cancer Panel (Invitrogen), and amplicon size was 75–125 bp. The amplicons were purified by Agencourt® AMPure® XP (Beckman Coulter, Miami, FL), and they were then end-repaired and ligated with Ion Xpress barcode adapters (Invitrogen). The median fragment size and concentration of the final library were detected by a BioAnalyzer using a High Sensitivity Chip (Agilent, Santa Clara, CA). The library was diluted to 10 pM by low TE, and 5 µL of the library was used for emulsion PCR reactions using Onetouch™ reagent kit. (Invitrogen); thereafter, the emulsion PCR product was enriched by Dynabeads® MyOne™ Streptavidin C1 beads (Invitrogen). The final enriched Ion spheres were mixed with a sequencing primer and polymerase, and loaded onto five 316 chips in total. Base calling was generated by the Torrent Suite 3.0 using tmap-f3 on the Ion Torrent server for further analysis. Bam and FASTQ files (alignment) were generated based on the base calling result, and were used to report the variant calling, including single nucleotide polymorphisms (SNPs) and insertions/deletions (INDELs).

### Statistical analyses

In the agreement analyses, the test performance was characterized by comparing the evaluable paired results between the PNA-LNA PCR clamp or Ion torrent PGM and direct sequencing. Positive percent agreement (PPA), negative percent agreement (NPA) and overall percent agreement (OPA) is calculated from the result of the each test. Agreement between direct DNA sequencing and other methodologies was also determined using κ statics. A χ^2^ test was used to assess the association between *EGFR* mutational status from each methodology and the tumor response to *EGFR* TKIs. All time-to-event outcomes were estimated using the Kaplan–Meier method and compared across groups with a log-rank test. All statistical tests were 2-sided, and statistical significance was defined as P<0.05. All analyses were performed using SPSS version 18.0 (SPSS Inc., Chicago, IL).

## Results

### Study design

Two distinct cohorts were examined in total with different methodologies for the detection of *EGFR* mutations. Since large amount of DNA was required for direct sequencing, PNA-LNA PCR clamp, and Ion Torrent PGM analysis, study cohort A was consisted of 57 fresh frozen NSCLC samples from 2 institutions running sample banks. Cohort B consisted of 42 FFPE NSCLC samples from 5 institutions. Since the DNA was obtained from stored FFPE and the amount of DNA from tumor tissue was small, the *EGFR* mutational status of cohort B was tested with direct sequencing and PNA-LNA PCR clamp. Clinical data regarding response to *EGFR* TKIs and survival were evaluated using the relevant clinical information of the patients. PNA-LNA PCR clamp was carried out in Cancer Research Institute at Korea University (Seoul, Korea). Direct sequencing and Ion Torrent PGM was carried out in Macrogen Inc. (Seoul, Korea).

### Comparison of sequencing methodologies: Detection of EGFR mutations by direct sequencing, PNA-LNA clamp PCR, and the Ion Torrent PGM

The primary objective of this study was to compare the analytic performance of PNA-LNA PCR clamp and Ion Torrent PGM with direct sequencing for detection of the predominant *EGFR* mutation. *EGFR* status was evaluated in all 57 samples in cohort A by using the three separate methodologies. *EGFR* mutations were identified in 16 (28.1%), 27 (47.4%), and 26 (45.6%) of 57 patients by direct sequencing, PNA-LNA PCR clamp, and Ion Torrent PGM, respectively.

The EGFR mutational status by the three methodologies and corresponding variant allele frequency (VAR) obtained from the Ion Torrent PGM was shown on Table 1. Direct sequencing detected the mutation of exon 21 (L858R) in 12 cases with VAR higher than 10% by Ion Torrent PGM. Although direct sequencing could not detect the case of CMC 72, who had 25.7% VAR, other 2 cases with VAR 2.74 and 5.04 were read as wild type by direct sequencing. Direct sequencing was particularly insensitive in detecting exon 19 deletion. Three cases with exon 19 deletion VAR higher than 50% were detected by direct sequencing, while 7 cases with VAR less than 50% were defined as wild type by direct sequencing. Overall 13(13/41, 31.7%) cases of wild type *EGFR* tumor by direct sequencing were identified as having mutation when tested either PNA-LNA PCR clamp or Ion Torrent PGM (Table 2).

**Table 1:** EGFR mutations status detected by direct sequencing, PNA-LNA PCR clamp, and Ion Torrent PGM accompanied with their variant frequencies and clinicopathologic features (n = 28).

ID	Sex/Age	Smoking history	Staging	Direct sequencing	PNA-LNA PCR clamp	Ion Torrent PGM	Variant Allele Frequency
KU2T	F/43	Never	IIA	Ex21(L858R)	Ex21(L858R)	Ex21(L858R)	12.03
KU8T	F/53	Never	IIB	Ex21(L858R)	Ex21(L858R)	Ex21(L858R)	31.27
KU11T	F/51	Never	IA	Ex21(L858R)	Ex21(L858R)	Ex21(L858R)	21.25
KU12T	F/51	Never	IIB	Ex21(L858R)	Ex21(L858R)	Ex21(L858R)	33.26
KU16T	F/60	Never	IIB	Ex21(L858R)	Ex21(L858R)	Ex21(L858R)	24.74
KU20T	F/64	Never	IB	Ex21(L858R)	Ex21(L858R)	Ex21(L858R)	10.35
KU23T	F/66	Ex-smoker	IIA	Ex21(L858R)	Ex21(L858R)	Ex21(L858R)	10.57
KU24T	F/66	Ex-smoker	IA	Ex21(L858R)	Ex21(L858R)	Ex21(L858R)	41.85
KU31T	F/72	Never	IIB	Ex21(L858R)	Ex21(L858R)	Ex21(L858R)	10.08
CMC57	F/60	Never	IA	Ex21 (L858R)	Ex21 (L858R)	Ex21(L858R)	21.37
CMC63	M/61	Ex-smoker	IA	Ex21 (L858R)	Ex21 (L858R)	Ex21(L858R)	31.31
CMC70	M/70	Ex-smoker	IA	Ex21 (L858R)	Ex21 (L858R)	Ex21(L858R)	23.22
CMC72	F/72	Never	IA	W	Ex21 (L858R)	Ex21(L858R)	25.7
CMC46	F/48	Never	IB	W	Ex21 (L858R)	W	0
CMC56	F/66	Never	IA	W	Ex21 (L858R)	Ex21 (L858R)	2.74
CMC59	M/76	Never	IB	W	Ex21 (L858R)	Ex21 (L858R)	5.07
CMC52	F/53	Never	IIIA	W	Ex21 (L858R)	W	0
					Ex19(2236–2250 del.)	Ex19(2236–2250 del.)	25.05
CMC55	F/81	Never	IA	W	Ex21 (L858R)	W	0
KU14T	F/63	Never	IIB	W	Ex19(2235–2249 del.)	Ex19(2235–2249 del.)	22.85
KU15T	F/63	Never	IV	W	Ex19(2235–2249 del.)	Ex19(2235–2249 del.)	41.38
KU27T	F/47	Never	IIIA	W	Ex19(2235–2249 del.)	Ex19(2235–2249 del.)	47.03
KU33T	F/48	Never	IB	W	Ex19(2235–2249 del.)	Ex19(2235–2249 del.)	49.8
CMC53	F/65	Never	IV	W	Ex19 (2235–2249 del.)	Ex19(2235–2249 del.)	36.28
CMC54	F/55	Never	IA	W	W	Ex19(2241–2255 del.)	22.58
CMC68	M/63	Current	IA	W	Ex19 (2235–2249 del.)	Ex19(2235–2249 del.)	25.57
KU10T	F/54	Never	IIIA	Ex19(2235–2249 del.)	Ex19(2235–2249 del.)	Ex19(2235–2249 del.)	62.59
KU17T	F/63	Never	IIA	Ex19(2235–2249 del.)	Ex19(2235–2249 del.)	Ex19(2235–2249 del.)	68.32
CMC74	M/72	Ex-smoker	IA	Ex19 (2236–2250)	Ex19 (2236–2250 del.)	Ex19 (2236–2250 del.)	85.91

**Table 2:** Comparison of the PNA-LNA PCR clamp with direct sequencing and Ion torrent PGM for detection of *EGFR* mutation.

PNA-LNA PCR clamp(Test Method)	Direct Sequencing (Reference Method)		
	Positive	Negative	Total
Positive, No.	16	11	27
Negative, No.	0	30	30
Total, No. (%)	16	41	57
PPA (95% CI)	16/16 = 100.00% (79.4–100.0)		
NPA (95% CI)	30/41 = 73.2% (65.1–73.2)		
OPA (95%CI)	16+30/57 = 80.7%		

Ion-Torrent PGM(Test Method)	Direct Sequencing (Reference Method)		
	Positive	Negative	Total
Positive, No.	16	10	26
Negative, No.	0	31	31
Total, No. (%)	16	41	57
PPA (95% CI)	16/16 = 100% (79.4–100.0)		
NPA (95% CI)	31/42 = 75.6% (67.6–75.6)		
OPA (95%CI)	16+31/57 = 82.5%		

Five cases showed different EGFR status either by PNA-LNA PCR clamp or Ion Torrent PGM. In three cases of *EGFR* mutations detected by Ion Torrent PGM, the mutations were identified on sequences other than predominant *EGFR* mutations, which was not designed be detected in PNA-LNA PCR clamp. The one of the 3 cases was known to be related with rare *EGFR* mutation of D761Y [17] on exon 19, and mutations of the other 2 cases were exon 19 mutations which were not to be clinically significant. To be accurate, because the mutations were designed not to be found on PNA-LNA PCR clamp originally, the interpretation of the 3 cases were not counted as discordant cases. In the other one case, *EGFR* mutation Ex21 (L858R) detected by PNA-LNA PCR clamp was not detected by Ion Torrent PGM. In a case (CMC52), both deletion on exon 19 and mutation on exon 21 was identified by PNA-LNA PCR clamp, however mutations on Ex21 (L858R) was not detected by Ion Torrent PGM. In CMC 55 case, mutation exon 21 was only detected by PNA-LNA PCR clamp.

For analysis of agreement between PNA-LNA PCR clamp PCR and Ion Torrent PGM, mutation positive was defined as the presence of the predominant oncogenic *EGFR* mutation type exon 19 deletions(2235–2249, 2239–2247, or 2236–2250) or the exon 21 L858R, as designed for PNA-LNA PCR clamp; all other valid non-predominant *EGFR* results and single nucleotide variations were considered mutation negative. For the 57 evaluable specimens, the positive percent agreement (PPA) between the PNA-LNA PCR clamp (test method) and direct sequencing (reference method) was 100.00%; negative percent agreement (NPA) was 73.2%; and overall percent agreement (OPA) was 80.7%. The positive percent agreement (PPA) between the Ion Torrent PGM (test method) and direct sequencing (reference method) was 100.00%; negative percent agreement (NPA) was 75.6%; and overall percent agreement (OPA) was 82.5%. PNA-LNA PCR clamp and the Ion Torrent PGM showed substantial concordance (κ = 0.965, *P*<0.001) to detect *EGFR* mutation. Mutational status between direct sequencing and PNA-LNA PCR clamp or the Ion Torrent PGM resulted in concordance between methodologies (κ = 0.605, *P*<0.001, and κ = 0.635, *P*<0.001).

### Correlation among EGFR mutational status, clinical response, and survival to treatment with EGFR TKIs

The other primary objective of this study was to evaluate clinical impact of *EGFR* mutations detected by PNA-LNA PCR clamp compared to direct sequencing. Scarcity of the available tissue and incomprehensive data regarding treatment restricted analysis of 6 patients in the cohort. The 4 cases with limitation only on response to EGFR TKI were included. In total, 36 out of 42 patients in cohort B were included to final analysis. All patients received *EGFR* TKIs (either erlotinib or gefitinib). The median age was 64.0±11.4 years (range 39–82) and the majority of patients had adenocarcinoma cell type (83.3%) and metastatic disease (67.6%). There was no statistically difference between patients with EGFR wild type versus mutant type tested by both direct sequencing and PNA-LNA PCR clamp (Table 3). Clinical characteristics of 6 patients with *EGFR*-wild type from direct sequencing in contrary to mutations detected from PNA-LNA PCR clamp are shown in Table 4.

**Table 3:** Clinicopathologic characteristics of patients with EGFR-TKI treatment.

	N (%)	EGFR mutation by direct sequencing		p-value	EGFR mutation by PNA-LNA PCR clamp		p-value
		(+)	(−)		(+)	(−)	
Total	36	12	24		18	18	
Sex							
Female	22(61.1)	9(75.0)	13(54.2)	0.227	13(72.2)	9(50.0)	0.171
Male	14(39.4)	3(25.0)	11(45.8)		5(27.8)	9(50.0)	
Age>65 yrs	16	4(33.3)	12(50.0)	0.343	5(27.8)	11(61.1)	0.092
Smoking status							
Never smoker	11(30.6)	2(18.2)	9(81.8)	0.271	3(17.6)	8(47.1)	0.067
Current or Ex-smoker	23(67.6)	9(81.8)	14(60.9)		14(82.4)	9(52.9)	
Unknown	2(5.6)						
Cell type							
Adenocarcinoma	30(83.3)	11(91.7)	19(79.2)	0.640	16(88.9)	14(77.8)	0.371
Non-adenocarcinoma	6(16.7)	1(8.3)	5(83.3)		2(11.1)	4(22.2)	
Clinical staging at initial diagnosis							
II	2(5.6)	1(8.3)	1(4.2)	0.539	1(5.6)	1(5.6)	0.322
III	10(27.8)	2(16.7)	8(33.3)		3(16.7)	7(38.9)	
IV	24(66.7)	9(75.0)	15(62.5)		14(77.8)	10(55.6)	
Previous therapy							
Curative Surgery or chemoradiation	14(38.9)	4(33.3)	10(41.7)	0.441	6(33.3)	8(44.5)	0.145
Palliative chemotherapy	19(52.8)	8(66.7)	11(45.8)		12(66.7)	7(38.9)	
None	3(8.3)	0	3(12.5)			3(16.7)	
ECOG status							
0 or 1	28(77.8)	10(83.3)	18(75.0)	0.571	14(50.0)	4(50.0)	1.00
2, 3 or 4	8(22.2)	2(16.7)	6(25.0)		14(50.0)	4(50.0)	
Types of EGFR TKIs							
Gefitinib	19(52.8)	6(50.0)	13(54.2)	0.813	10(55.6)	9(50.0)	0.738
Erlotinib	17(47.2)	6(50.0)	11(45.8)		8(44.4)	9(50.0)	

**Table 4:** Distribution of EGFR mutations detected by direct sequencing, PNA-LNA PCR clamp in discordant cases (6 cases).

Case No,	Histo type	Sex/Age	Smoking	Initial Stage	Specimen	TKI response	PFS(mo)	OS(mo)	Direct sequencing	PNA-LNA PCR clamp
1	SQ	M/51	Current	IV	Wedge biopsy	SD	0.10	2.95	WT	Ex21(L858R)
2	AD	F/74	Never	IIa	Lobectomy	PR	3.25	13.84	WT	Ex19(2235–2249del)
3	AD	F/39	Never	IIIb	Pleural biopsy	SD	36.79	56.92	WT	Ex21(L858R)
4	AD	M/53	Never	IV	FNA	SD	21.21	27.77	WT	Ex21(L858R)
5	AD	F/56	Never	IV	Lobectomy	Unknown	Unknown	Unknown	WT	Ex21(L858R)
6	AD	F/64	Never	IV	FNA	PR	31.74	39.54	WT	Ex19(2235–2249del) Ex21(L858R)

*EGFR* Mutations were identified in 33.3% (12/36) and 50% (18/36) of the 36 patients by direct sequencing and PNA-LNA PCR clamp, respectively. Regarding the responses to EGFR TKIs of the 12 patients with positive mutations as detected by direct sequencing, there was 1 complete response, 8 partial responses (PR), and 0 stable diseases (SD) as the best response, for a total response rate and disease control rate of 75% (9/12). Regarding the responses to EGFR TKIs of the 18 patients with positive mutations as detected by PNA-LNA PCR clamp, except a case which is defective in response evaluation, there was 1 CR, 10 PR, and 3 SD as the best response, for a total response rate of 64.7% (11/17) and disease control rate of 82.4% (14/17). Patients with *EGFR* mutations tested by PNA-LNA PCR clamp did not show a significantly higher response rate than did patients with the wild-type *EGFR* (*P* = 0.470) when treated with EGFR TKIs. A similar result was observed when *EGFR* mutations were tested with direct sequencing (*P* = 0.074).

**Figure 1:**
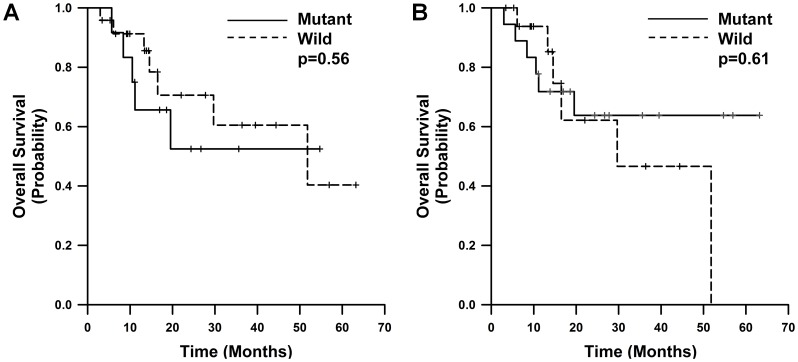
Kaplan-Meier analysis of OS by EGFR status detected (A) on PNA-LNA PCR clamp and (B) on direct sequencing.

The median overall survival (OS) for all patients was 51.8±22.86 months in a median follow-up time of 73.8±32.26 months. The OS for each of the groups was 34.39±6.81 months in patients with EGFR mutations by direct sequencing, 44.10±6.34 months by PNA-LNA PCR clamp. Mutational status detected by 2 methods did not significantly predict overall survival.(*P* = 0.582 for direct sequencing, *P* = 0.736 for PNA-LNA PCR clamp) However, curves showed a greater difference when the mutation was detected by PNA-LNA PCR clamp as compared to mutations detected by direct sequencing (Fig. 1).

**Figure 2:**
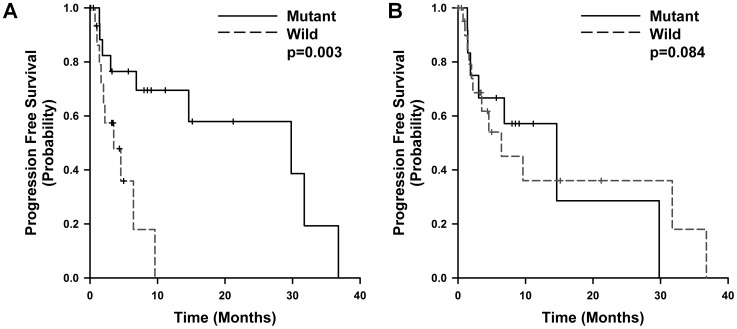
Kaplan–Meier analysis of PFS by EGFR status detected (A) on PNA-LNA PCR clamp and (B) on direct sequencing.

The median progression free survival (PFS) for all patients was 6.85±3.19 months. The median PFS in each of the groups was 14.31±3.91 months in patients with EGFR mutations in the direct sequencing group, 21.61±3.9 months in the PNA-LNA PCR clamp group. A Kaplan-Meier survival analysis showed that patients with mutations detected by PNA-LNA PCR clamp showed a significantly longer PFS than did those with wild-type *EGFR (Log rank, P = 0.003)* (Fig. 2). Mutational status detected by direct sequencing did not significantly predicted progression free survival. In the Cox regression analysis, only ECOG performance status (PS) remained an independent predictor of PFS and OS (HR = 3.854, *P* = 0.029).

## Discussion

To our knowledge, this is the first comparison of PNA-LNA PCR clamp and direct sequencing to assess potential predictive biomarkers of response using FFPE tumor samples. Previous reports described the use of next-generation sequencing techniques to investigate *EGFR* mutations without therapeutic relevance [18], [19].

In this study, we showed that detection of *EGFR* mutations by direct sequencing is less sensitive than PNA-LNA PCR clamp and Ion Torrent PGM. Each method revealed *EGFR* mutations in 11(26.2%) patients by PNA-LNA PCR clamp and 13(31.0%) patients by Ion Torrent PGM out of 42 wild type patients by direct sequencing in cohort A and another 6 wild type patients by direct sequencing in cohort B (6/36, 16.7%) revealed mutation positive by PNA-LNA PCR clamp, which could be missed the opportunities of treatment with EGFR-TKIs. Particularly, direct sequencing is less sensitive in the cases of low VAR. Direct sequencing detected mutations of exon 21(L858R) with mean VAR 22.85, which is similar to the known sensitivity of the direct sequencing. However, detecting deletions on exon 19 by direct sequencing required threshold of VAR more than 50. Considering exon 19 mutations are as frequent as exon 21(L858R) and related with better response to EGFR TKIs compared to exon 21 mutation [20], failure to detect mutations on exon 19 is definite disadvantage of direct sequencing. Though there are controversies on optimal mutant allele burden cut-off [21], VAR was evaluated in the context to guide quantitative value of mutant allele frequency. More study is required for to determine cut-off value of clinically significant quantity of mutation.

Although detection of *EGFR* mutational status did not differ significantly between PNA-LNA PCR clamp and the Ion Torrent PGM, inconsistency in the types of *EGFR* mutations was observed between PNA-LNA PCR clamp and the Ion Torrent PGM. The clinical significance of double mutations is still uncertain, but the aforementioned cases suggests the possibility of heterogeneity within the tumor cells [22]. For application of NGS in clinic, specific technical challenges including the use of heterogeneous tumor samples and the use of small amounts of degraded and fixative-affected DNA are needed [23]. Therefore, further study is needed to find out which of the methods is best for manipulating FFPE clinical material with acceptable cost and turnaround times for clinical decision making in the use of NGS. Despite of exceptionally high specificity and sensitivity, PNA-LNA PCR clamp has limitation in that LNA probes are designed to bind specific mutation sequence, which means the test cannot identify minor alterations on other sequence. NGS such as Ion Torrent PGM could serve to fill this gap in identifying new target for cryptic dysregulated cellular pathways and novel therapy.

Given that direct sequencing has limited sensitivity, those sensitive methods to predict the response of *EGFR* TKIs have not been systemically compared and it is not clear which test provides the best performance [10], [13], [24]. In this study, we have shown that not only patients with tumors harboring *EGFR* mutations detected by direct sequencing but also patients with *EGFR* mutations detected by PNA-LNA PCR clamp benefit from EGFR-TKI in terms of progression free survival. The limitations of this study could be considered according to a number of perspectives: firstly, the small number of samples could affect outcomes not by biomarker but by the characteristics of the subset of patients. Secondly, retrospective nature, and absence of an unaffected and balanced control group in this study might limit the value of predictability of the biomarker, EGFR. Lastly, use of archival tissue in the test methodologies could have resulted in changes of biomarker status over time and treatment.

As a conclusion, we have shown that patients harboring low EGFR mutation could benefit from EGFR TKI therapy. The discrepancies among those three methodologies are generally matched with the fact that the sensitivity of direct sequencing is suboptimal for many clinical tumor samples, in that mutant DNA alleles must comprise over 25% of the total DNA signals to be readily detected. Logically, optimized diagnosis through more sensitive bioassay could have major consequences in terms of cost-effectiveness by further rationalizing the selection of candidate for EGFR TKI. Though this study was conducted in a small number of cases, the results suggest that both PNA-LNA PCR clamp and the Ion Torrent PGM are highly sensitive procedures compared to direct DNA sequencing, and are useful screening tools for the detection of *EGFR* mutations in clinical practice. Further studies are needed to determine whether differences in *EGFR* mutation status detected using methods with different sensitivities are associated with treatment response to *EGFR* TKIs in lung carcinoma.
